# Sensor Signal and Information Processing II

**DOI:** 10.3390/s20133751

**Published:** 2020-07-04

**Authors:** Wai Lok Woo, Bin Gao

**Affiliations:** 1 Department of Computer and Information Sciences, Northumbria University, Newcastle upon Tyne NE1 8ST, UK; 2 School of Automation Engineering, University of Electronic Science and Technology of China, Chengdu 610054, China; bin_gao@uestc.edu.cn

**Keywords:** sensors, phase arrays, signal processing, image processing, 3D processing, deep learning, machine learning, compressive sensing, wireless communication

## Abstract

This Special Issue compiles a set of innovative developments on the use of sensor signals and information processing. In particular, these contributions report original studies on a wide variety of sensor signals including wireless communication, machinery, ultrasound, imaging, and internet data, and information processing methodologies such as deep learning, machine learning, compressive sensing, and variational Bayesian. All these devices have one point in common: These algorithms have incorporated some form of computational intelligence as part of their core framework in problem solving. They have the capacity to generalize and discover knowledge for themselves, learning to learn new information whenever unseen data are captured.

## 1. Introduction

Smart sensors are revolutionizing the world of system design in everything from sports cars to assembly lines. These new sensors have abilities that leave their predecessors in the dust. They not only measure parameters efficiently and precisely, but they also have the ability to enhance and interrupt those measurements, thereby transforming raw data into truly useful information. The concept of the smart sensor was first introduced by NASA in the process of developing a spaceship and formed as a product in 1979. Smart sensors have the ability to automatically calibrate, compensate, and collect data. Their capability determines that the smart sensors have high accuracy and resolution, high stability and reliability, and good adaptability. Compared with traditional sensors, they have a high performance price ratio. Early smart sensors are processed and converted from the output signal of the sensor to the microprocessor for operation. In the 1980s, the smart sensor mainly focused on microprocessors, and integrated the sensor signal conditioning circuit, microelectronic computer memory, and interface circuit to a chip, so that the sensor has certain artificial intelligence (AI). Currently, intelligent measurement technology has been further improved so that the sensor can achieve miniaturization and has the function of self-diagnosis, and to work co-creatively with human and AI [1].

## 2. Contributions

This Special Issue includes twenty-four works focused on sensor signals and information processing based on diverse technologies for different applications

Machine learning techniques have recently been introduced for phishing detection. Phishing is a very common social engineering technique. The attackers try to deceive online users by mimicking a uniform resource locator (URL) and a webpage. Traditionally, phishing detection is largely based on manual reports from users. Wei et al. [2] have proposed an accurate and low-cost phishing detection sensor by exploring deep learning techniques. In nonlinear filtering, Lou et al. [3] have introduced an algorithm based on alpha-divergence, which uses the exponential family distribution to approximate the actual state distribution and the alpha-divergence to measure the approximation degree between the two distributions. In this way, it provides more choices for similarity measurement by adjusting the value of alpha during the updating process of the equation of state and the measurement equation in non-linear dynamic systems.

In compressive sensing, Wang et al. [4] have presented a regularized weighted smoothed *L*_0_-norm minimization method for underdetermined blind source separation. The algorithm is constructed under the gradient descent method, in which the update process adopts the regularization mechanism to enhance the de-noising performance. A weighted smoothed function is also proposed to promote sparsity and provide the guarantee of robust and accurate signal recovery. Compressed sensing current mapping offers additional advantages, such as high speed without the use of complicated experimental layouts or lock-in amplifiers. Koutsourakis et al. [5] have demonstrated that the sparsity of the patterns used for compressive sampling can be controlled to achieve significant signal amplification of at least two orders of magnitude, while maintaining or increasing the accuracy of measurements. In the existing stochastic gradient matching pursuit algorithm, the preliminary atomic set includes atoms that do not fully match the original signal. This weakens the reconstruction capability and increases the computational complexity. Zhao et al. [6] have proposed an algorithm that uses the weak selection threshold method to select the atoms that best match the original signal from the block sensing matrix and completes the expansion of the preliminary atomic set.

Sensors data need to be processed after acquisition to remove noise and extract relevant information. When the sensor is a network node and acquired data are to be transmitted to other nodes, the amount of generated data from multiple nodes can overload the communication channel. The reduction in generated data implies the possibility of lower hardware requirements and less power consumption for hardware devices. In this context, Barrios-Aviles et al. [7] have proposed a filtering algorithm which reduces the generated data from event-based sensors without the loss of relevant information. It is a bioinspired filter and event data are processed using a structure resembling biological neuronal information processing. The filter is fully configurable from a “transparent mode” to a very restrictive mode. Chianphatthanakit et al. [8] have presented a compression algorithm applied to the domain of wireless sensor nodes in which energy use is one of the most important aspects. In biomedical sensing, electrocardiogram signal analysis is based on detecting a fiducial point consisting of the onset, offset, and peak of each waveform. The accurate diagnosis of arrhythmias depends on the accuracy of fiducial point detection. Lee et al. [9] have proposed a method that minimizes the number of candidate samples for fiducial point detection and emphasizes these samples’ feature values to enable reliable detection, while still sensitive to the morphological changes of various QRS complexes by generating an accumulated signal of the amplitude change rate between vertices as an auxiliary signal. Sensors based on tunable diode laser absorption spectroscopy have the advantages of high sensitivity, high stability, high selectivity and fast response, and have been widely applied in atmospheric environmental monitoring. Zhang et al. [10] have conducted a review and compared some effective algorithms using representative experiments to evaluate the performance both qualitatively and quantitatively.

In imaging, Zhou et al. [11] have presented a method for surface reconstruction from image-based point clouds. In particular, the authors introduce a new visibility model for each line of sight to preserve scene details without decreasing the noise filtering ability. Color filter array (CFA) demosaicing has established itself as the de facto standard method of acquiring multi-dimensional color images. General demosaicing would be defined as the reconstruction of a multi-dimensional color signal from an inherently single-dimensional array of sensors. In [12], Stojkovic et al. have tested the influence of Bayer-like CFA patterns with the same sampling ratio as the Bayer CFA, considering them to be sufficient to show that the difference in quality performance between different CFA designs decreases with the increasing power of demosaicing algorithms.

Research on data-driven fault diagnosis methods has received much attention in recent years. The deep belief network is a commonly used deep learning method for fault diagnosis. In the past, researchers used Deep Belief Network (DBN) to diagnose gear pitting faults but the diagnosis result was not good with continuous time domain vibration signals as direct inputs into DBN. Li et al. [13] have proposed a method by stacking a spare autoencoder and Gauss-binary restricted Boltzmann machine for early gear pitting faults diagnosis with raw vibration signals as direct inputs. The stacking spare autoencoder layer is used to compress the raw vibration data while the Gaussian-Bernoulli Restricted Boltzmann Machine (GBRBM) layer is used to effectively process continuous time domain vibration signals. Bearings are critical parts of rotating machines, making bearing fault diagnosis based on signals a research hotspot through the ages. In real application scenarios, bearing signals are normally non-linear and unstable, and thus difficult to analyze in the time or frequency domain only. Wu et al. [14] have proposed a method based on the self-adaptive spectrum analysis diagnosis framework to solve these problems. Diagnosing the fault of rolling element bearings and ensuring normal operation is essential. However, the faults of rolling element bearings under variable conditions and the adaptive feature selection have rarely been discussed. Wang et al. [15] have presented a practical method based on the Mahalanobis–Taguchi system to put forward the disposal of the fault under variable conditions. Hou et al. [16] have proposed a variational Bayesian-based adaptive shifted Rayleigh filter to improve the tracking performance in bearings-only target tracking in the presence of clutter. The target state and the clutter probability are jointly estimated to account for the uncertainty in clutter probability.

In ultrasonic nondestructive testing, the detection of flaw echoes in backscattered signals can be challenging due to the existence of backscattering noise and electronic noise. Feng et al. [17] have proposed an empirical mode decomposition methodology for flaw echo enhancement. Guo et al. [18] have presented a fault diagnosis method based on a continuous wavelet transform scalogram and a Pythagorean spatial pyramid pooling convolutional neural network for fault diagnosis of rotating machinery. Xu et al. [19] have introduced a monitoring methodology of dam deformation by taking the advantages of terrestrial laser scanning, which is capable of capturing geometric information at a huge amount of points over an object in a relatively fast manner. In robotics control, robot manipulators are often required to quickly detect collisions to limit damage due to physical contact. Traditional model-based detection methods in robotics are mainly concentrated on the difference between the estimated and actual applied torque. Min et al. [20] have proposed a model independent collision detection method based on the vibration features generated by collisions.

In wireless communication, the time difference of arrival based on a group of sensor nodes with known locations has been widely used to locate targets. Wu et al. [21] have presented a hybrid firefly algorithm method, combining the weighted least squares and firefly algorithms which reduces the computation as well as achieves high accuracy. Liu et al. [22] have proposed a switched-element direction finding system-based direction of arrival estimation method for un-cooperative wideband orthogonal frequency division multi-linear frequency modulation radar signals. Li et al. [23] have presented a fine delay scheduling architecture in the multi-group scanning of an ultrasonic phased arrays system where the diversity of echo data in multi-group scanning and the number of focal laws are considered, and the multi-group scan problem is modelled by a linear equation. Liu et al. [24] have proposed an iterative high-accuracy method based on fractional Fourier transform and fractional autocorrelation interpolation to improve the parameter estimation performance of uncooperative orthogonal frequency division multi-linear frequency modulation radar signals. In practical applications, the assumption of omnidirectional elements is not effective in general, which leads to direction-dependent mutual coupling. Under this condition, the performance of traditional calibration algorithms suffers. Qi et al. [25] have presented a self-calibration method based on time–frequency distributions in the presence of direction-dependent mutual coupling which outperforms the existing algorithms.
